# Leveraging multi-omics data to infer regulators of mRNA 3’ end processing in glioblastoma

**DOI:** 10.3389/fmolb.2024.1363933

**Published:** 2024-08-12

**Authors:** Aleksei Mironov, Lorenzo Franchitti, Shreemoyee Ghosh, Marie-Francoise Ritz, Gregor Hutter, Michele De Bortoli, Mihaela Zavolan

**Affiliations:** ^1^ Biozentrum, University of Basel, Basel, Switzerland; ^2^ Department of Clinical and Biological Sciences, University of Turin, Turin, Italy; ^3^ Department of Biomedicine, University of Basel, Basel, Switzerland

**Keywords:** glioblastoma, TCGA, ENCODE, RBP, alternative polyadenylation

## Abstract

Alterations in mRNA 3’ end processing and polyadenylation are widely implicated in the biology of many cancer types, including glioblastoma (GBM), one the most aggressive tumor types. Although several RNA-binding proteins (RBPs) responsible for alternative polyadenylation (APA) were identified from functional studies in cell lines, their contribution to the APA landscape in tumors *in vivo* was not thoroughly addressed. In this study we analyzed a large RNA-seq data set of glioblastoma (GBM) samples from The Cancer Genome Atlas (TCGA) to identify APA patterns differentiating the main molecular subtypes of GBM. We superimposed these to RBP footprinting data and to APA events occurring upon depletion of individual RBPs from a large panel tested by the ENCODE Consortium. Our analysis revealed 22 highly concordant and statistically significant RBP-APA associations, whereby changes in RBP expression were accompanied by APA in both TCGA and ENCODE datasets. Among these, we found a previously unknown PTBP1-regulated APA event in the PRRC2B gene and an HNRNPU-regulated event in the SC5D gene. Both of these were further supported by RNA-sequencing data of paired tumor center-periphery GBM samples obtained at the University Hospital of Basel. In addition, we validated the regulation of APA in PRRC2B by PTBP1 in siRNA-knockdown and overexpression experiments followed by RNA-sequencing in two glioblastoma cell lines. The transcriptome analysis workflow that we present here enables the identification of concordant RBP-APA associations in cancers.

## Introduction

The processing of eukaryotic mRNAs involves 3′ end cleavage and addition of a polyadenosine (poly(A)) tail. These steps are carried out by the 3′ end processing complex, which contains around 20 core proteins (Gruber and Zavolan, 2019). Most human genes express multiple mRNA isoforms, which differ in their use of transcription start site, 3′ end processing site and/or internal exons. Variation in the choice of 3′ end processing sites (alternative polyadenylation, APA), gives rise to mRNA isoforms of different lengths, and often the difference is only in the length of 3′ untranslated regions (3′UTRs). As 3′UTRs interact with many proteins and protein complexes, the consequences of 3′UTR APA are many, ranging from changes in mRNA stability and localization to changes in the localization of the encoded protein (Mayr, 2019). 3′UTR APA accompanies changes in cellular state: 3′UTRs become longer during cell differentiation (Ji et al., 2009) and shorter in tumors (Xia et al., 2014), where most cell types have an altered polyadenylation landscape with shortened 3′UTRs (Burri and Zavolan, 2021).

Since the realization that 3′UTRs undergo systematic shortening in cancer cells (Mayr and Bartel, 2009), there is great interest in identifying the responsible regulators. An important candidate is CPSF5 (also known as CFIm25), a protein of 25 kDa that takes part, as a dimer, in the mammalian cleavage factor I (CFIm) complex (Yang et al., 2011). This complex typically binds to UGUA motifs located ∼40 nucleotides upstream of the 3′ end processing site (also known as polyadenylation site, poly(A) site or PAS) (Yang et al., 2010), promoting their processing. The knockdown of CFIm leads to the use of coding-region proximal poly(A) sites (pPAS) and global shortening of 3′UTRs (Gruber et al., 2012; Martin et al., 2012), which is why this protein has been actively researched in the context of cancers and ultimately implicated in the observed APA (reviewed in (Masamha, 2023)). Since these initial studies, a variety of RNA-binding proteins (RBPs) have emerged as regulators of APA and often of splicing as well. HNRNPC is one of these regulators, best known for its role in suppressing the inclusion of Alu elements in mRNAs (Zarnack et al., 2013), and more recently found to also repress the usage of PAS containing in their vicinity the polyuridine binding motif of HNRNPC (Gruber et al., 2016). In a previous study of data from The Cancer Genome Atlas (TCGA), we found that 3′UTR shortening also occurs in glioblastoma (GBM), and we have associated this APA pattern with the increased expression of the polypyrimidine tract binding protein 1 (PTBP1), which has much higher expression in GBM compared to normal tissue (Gruber et al., 2018).

Given the interest in APA in cancers and also the availability of multi-omics data not only from cancers but also pertaining to the impact of RBPs on RNA processing, we have developed an approach to identify individual regulatory relationships between PAS and RBPs in a disease context. We have applied this approach to GBM, a tumor type where APA has been studied before, thereby providing us with a good basis for evaluating the efficiency of the approach in identifying regulators of APA. Our results reveal a number of RBPs that have systematic association with 3′UTR shortening and lengthening including CFIm and HNRNPC. In GBM, we identify significant PAS-RBP associations that are consistent with RBP perturbation data. These associations involve a variety of RBPs, and we highlighted the examples of HNRNPU-SC5D and PTBP1-PRRC2B. The approach is general, and we expect that its application to other cancers will accelerate the discovery of key factors responsible for altered gene expression patterns in cancers.

## Methods

### RNA-sequencing data download and processing

The GRCh38 (hg38) assembly of the human genome along with the comprehensive gene annotation (v42) was downloaded from the GENCODE Consortium website (Frankish et al., 2021). Annotation was additionally supplemented with the non-coding genomic elements from RNAcentral portal (Consortium, 2021).

Genomic alignments (BAM format) of short paired-end reads from 150 glioblastoma RNA-seq IDH-WT samples of the TCGA-GBM project classified into three GBM subtypes (Wang et al., 2018) were obtained from the GDC portal (accession number phs000178.v11.p8). In addition, we required that all analyzed samples have defined tumor purity and survival information, which limited the final scope to 122 TCGA-GBM samples. The results of 472 RBP depletion experiments followed by paired-end RNA-seq including 440 shRNA-mediated knockdown and 32 CRISPR-mediated knockout experiments were downloaded from the ENCODE portal in BAM format (Van Nostrand et al., 2020). The list of all samples used in the study is presented in Supplementary Table S1.

All RNA-seq samples were identically processed. BAM files were converted to fastq files and then re-aligned to the hg38 assembly of the human genome using STAR-2.7.8a aligner in one-pass mode without supplying the annotation file and otherwise default parameters, as was previously done (Wilks et al., 2021). Samtools v1.18 (Li et al., 2009) was used to extract only uniquely mapped reads present in not more than 10 duplicates.

### RNA sequencing of tumor center-periphery samples

Human adult GBM tissue samples were obtained from the Neurosurgical Clinic of University Hospital of Basel, Switzerland, in accordance with the Swiss Human Research Act and institutional ethics committee (EKNZ 02019-02358). All patients gave written informed consent for tumor biopsy collection and signed a declaration permitting the use of their biopsy specimens in scientific research, including storage in our brain tumor biobank (Req-2019-00553). All patient-identifying information was removed, and tissues were coded for identification. Tumor samples from contrast-enhancing center and non-enhancing periphery according to intraoperative neuronavigation were snap frozen in the operating theater and stored in liquid N2 until RNA extraction. Tumor methylation subtyping from native genomic DNA was performed as described by Capper et al. using the Illumina Infinium Epic array (PMID 29539639).

RNA was extracted from four tumor center-periphery sample pairs using the AccuPure Tissue RNA Mini Kit. All samples were checked to have RIN number >6.5. Library preparation was performed, starting from 100 ng total RNA, using the TruSeq Stranded mRNA Library Kit (Cat# 20020595, Illumina, San Diego, CA, United States) and the TruSeq RNA UD Indexes (Cat# 20022371, Illumina, San Diego, CA, United States). 15 cycles of PCR were performed and the samples were sequenced on an Illumina NextSeq 500 instrument to obtain 38 bp paired-end reads. Reads in FASTQ format were mapped to the hg38 genome and further processed together with other public RNA-seq samples used in the study, using the same workflow (see above).

### Cell culture, transfections, and RNA-sequencing of glioblastoma cell lines

For the experimental validation of PTBP1’s role in glioblastoma cell lines, LN18 cells were cultured on D6546 medium (0.11 g/L NaP) supplemented with 4 mM L-glutamine, 5% FCS and 1X P/S. U-87 MG cells were grown on D6546 medium (0.11 g/L NaP) supplemented with 2 mM L-glutamine, 10% FCS and 1X P/S. For the knockdown of PTBP1, LN18/U-87 MG cells were seeded at a density of 25% in six-well plates. Following a 24 h incubation, at around 50% confluency, siRNAs against PTBP1 (siPOOLs from siTOOLs BIOTECH) were incubated with Lipofectamine RNAiMAX (Invitrogen) and added to the wells. Real-time qPCR (qRT-PCR) analyses were carried out to quantify PTBP1 expression on the StepOnePlus™ Real-Time PCR System. Specific primers used for detection of PTBP1 were.

PTBP1 FP: CCAAGTTCGGCACAGTGTTG.

PTBP1 RP: TATACCAGGTGCACCGAAGG.

Samples were analyzed in PCR triplicates from three biological replicates. The expression of PTBP1 protein were probed with the respective antibodies, Cell Signaling #57246, Cell Signaling #79940 and Cell Signaling #4799 at 1:1,000 dilution using the standard Western blotting protocol.

Total RNA was quality-checked and 200 ng total RNA was used for library preparation with the TruSeq Stranded mRNA Library Prep Kit High Throughput (Cat# RS-122-2103, Illumina, San Diego, CA, United States). Libraries were quality-checked on the Fragment Analyzer (Advanced Analytical, Ames, IA, United States) using the Standard Sensitivity NGS Fragment Analysis Kit (Cat# DNF-473, Advanced Analytical). Samples were sequenced using the Illumina NovaSeq 6,000 sequencing system with 50 bp paired-end reads.

### Quantification of tandem poly(A) sites usage

The polyAsite atlas (Herrmann et al., 2020) of the human genome (assembly version GRCh38.96) was downloaded in BED format. Tandem poly(A) sites within the terminal exons (TEs) were extracted with custom scripts. A modified PAQR workflow (Gruber et al., 2018) was used to extract the matrix of raw read counts supporting distinct poly(A) sites across all analyzed RNA-seq samples. Briefly, the workflow modifications included the calculation of the median instead of mean coverage level supporting the poly(A) site usage, and the omission of the step that ensured that the used PAS was identical to the best break point in the corresponding terminal exon region. We used the following stringent input parameters that allow the quantification of tandem poly(A) sites separated by the genomic distance of at least 1 kb, of which 700 nt region is reserved for the calculation of the median coverage level, and 300 nt is the region directly upstream from the poly(A) site where a coverage drop is expected (values set on the basis of mRNA fragment size targeted during sample preparation): PAQ_coverage_downstream_extension 700, PAQ_min_distance_start_to_proximal 1,020, PAQ_min_length_mean_coverage 700, PAQ_min_mean_exon_coverage 1, PAQ_distal_downstream_extension 700, PAQ_max_mean_coverage 50, PAQ_cluster_distance 1,000, PAQ_upstream_cluster_extension 320, PAQ_coverage_mse_ratio_limit 0.8, PAQ_fragment_length 320. We obtained quantification for 4,935 PAS within 1,982 terminal exons. In each terminal exon, we defined the most 3′-end-adjacent PAS as distal (dPAS), and other sites as proximal (pPAS). Hence, 2,953 PAS were termed proximal.

The relative usage of PAS within the terminal exon (PAU) was estimated as the number of reads supporting the PAS as a fraction of the total number of reads supporting all PAS within the terminal exon.

To account for the library size and the noise from low expression intensity that systematically confound the estimates of relative usage of alternative transcript isoforms (Kakaradov et al., 2012; Mironov et al., 2021), we used the following procedure to estimate the usage score of a poly(A) site (PAU score) relative to other poly(A) sites within the terminal exon. First, given the number of raw reads supporting a poly(A) site (M) and the total number of reads supporting all the sites at the terminal exon (N), we perform two binomial tests to obtain the following *p*-values:

${pval}_{1}=p\left(m>M\;|\;N,prob=0.5\right),{pval}_{2}=p\left(m<M\;|\;N,prob=0.5\right)$

We then calculate the score as: $score=\left(-1\right)\log_{10}{pval}_{1}-\left(-1\right)\log_{10}{pval}_{2}$

Thus, the score ranges from - ∞ to + ∞ and reflects the propensity of the PAS to yield a major (score ➝ +∞) or minor (score ➝ -∞) transcript isoform, controlling for the noise due to low read counts (Supplementary Figure S1A). Next, we calculated the size factors according to DESeq2 methodology (Love et al., 2014). Namely, each row of the expression matrix with samples in columns and poly(A) sites in rows was divided by the row median. The size factor ${sf}_{k}$ of the sample *k* was estimated as the median of the *k*th column. Then, for each poly(A) site, we fitted a quantile regression with the library size being the explanatory variable and the absolute value of the score being the response variable (an example for one poly(A) site is shown at Supplementary Figure S1B). We then used the positive residuals as library-size-controlled absolute score values, while negative residuals were zeroed. Each positive value was then multiplied by +1 or −1 to maintain the sign of the original score. The obtained values thus reflect the relative magnitudes of PAS usage controlled for library size and later referred to as PAS scores.

To analyze differential alternative polyadenylation (dAPA) between conditions, we calculated median PAU values for each condition and the difference of them for each PAS (∆ PAU, for proximal and distal it is denoted as ∆ pPAU and ∆ dPAU, respectively). By using the sample-level PAU levels as weights of PAS positions and comparing the condition-specific median levels, we adjusted ∆ PAU signs to ensure that higher PAU values of proximal PAS always correspond to shortening of the terminal exons, while higher PAU of distal PAS always correspond to lengthening. To evaluate the significance of ∆ PAU values, we employed different strategies as suited for a particular experimental setups. In ENCODE RBP depletion experiments, we ran DEXSeq (Anders et al., 2012) with default parameters individually in each experiment. We discarded experiments in which dispersion trend was not well captured, and corrected *p*-values for multiple-testing of many PAS using Benjamini–Hochberg approach (Benjamini and Hochberg, 1995). This yielded 0 (minimum) - 158 (maximum) significant dAPA events per experiment, with the median being 6.5 dAPA events. The same approach appeared incorrect for comparisons of TCGA-GBM subtypes, in which each condition comprises dozens of samples, as was evidenced from diagnostic dispersion plots (data not shown). Indeed, differential expression analysis between large cohorts of human population samples with DESeq package (Love et al., 2014), on which DEXseq is largely based, was recently reported to be prone to false positives (Li et al., 2022). The same study recommended using instead Mann-Whitney sum-of-ranks test (also known as Wilcoxon test) for the difference of medians. To adapt this recommendation for dAPA analysis, for each PAS independently, we fitted the following quantile regression model, similarly to a previous work (Mironov et al., 2021):

$r_{PAS}={\;\beta_{0}r}_{other}+\beta_{1}D_{cond}r_{other}$ where $r_{PAS}$ is the library-size normalized number of reads supporting the PAS, $r_{other}$ is the library-size normalized total number of reads supporting other PAS of the terminal exon, $D_{cond}$ is a dummy variable equal to zero in one condition and to one in another condition. An example of a fitted model for a proximal PAS in CHST11 gene is shown at Supplementary Figure S1C. We took the maximum of *p*-values from *t*-tests on β coefficients (Greene, 2008), and corrected them for multiple testing of many PAS using Benjamini–Hochberg approach (Benjamini and Hochberg, 1995). Thus we ensured that there is expected significant association of $r_{PAS}$ with the host gene expression (captured by $\beta_{0}$ ) and there is significant dependence on the condition (captured by $\beta_{1}$ ). In fact, median PAU values in each of the conditions can be computed from the fitted values of β coefficients: ${PAU}_{condition\;0}=\frac{{\hat{\beta }}_{0}}{1+{\hat{\beta }}_{0}}{,PAU}_{condition\;1}=\frac{{\hat{\beta }}_{0}+{\hat{\beta }}_{1}}{1+{\hat{\beta }}_{0}+{\hat{\beta }}_{1}}$ .

Thus, ∆ PAU can be reformulated as: $∆PAU=\frac{{\hat{\beta }}_{0}+{\hat{\beta }}_{1}}{1+{\hat{\beta }}_{0}+{\hat{\beta }}_{1}}-\frac{{\hat{\beta }}_{0}}{1+{\hat{\beta }}_{0}}$

Finally, in the identification of significant associations between PAS usage and the expression of RBPs, we compared median PAS scores in cohorts of high RBP expression and low RBP expression using Mann-Whitney sum-of-ranks test followed by Benjamini–Hochberg correction for multiple testing (Benjamini and Hochberg, 1995).

### Quantification of gene expression

Expression of protein-coding genes was evaluated from respective coding regions (CDS elements in the annotation) present in most annotated transcript isoforms. More precisely, for each protein-coding gene, we splitted the spanned genomic region into bins by the number of overlapping coding transcripts, and took the union of the bins having maximum overlapping transcripts. Thus produced custom GTF annotation file was as input to FeatureCounts package (Liao et al., 2014) to obtain per gene raw fragment counts across samples, with the following parameters: featureCounts -p -O --fraction -Q 255 -s 2 -B -C -P -d 0 -D 10000000000000 -t exon -g gene_id. Obtained raw values were then normalized by variance-stabilizing transformation (VST) within the DESeq package (Love et al., 2014).

To evaluate differential gene expression in ENCODE RBP perturbation experiments, DESeq2 package (Love et al., 2014) with default parameters was utilized independently for each experiment, obtained *p*-values were corrected for multiple-testing of many genes using Benjamini–Hochberg approach (Benjamini and Hochberg, 1995). In other analyses, Mann-Whitney sum-of-ranks test was utilized.

### Quality control of RNA-seq samples

Uniquely mapped low-duplication reads (see above) were analyzed with FastqC (https://www.bioinformatics.babraham.ac.uk/projects/fastqc/) to estimate median per-base sequence quality and other quality metrics. Median transcript integrity number (TIN score) (Wang et al., 2016) was calculated for each sample. The fractions of fragments mapped to each transcript biotype, as well as intergenic and antisense segments, are further important RNA-seq quality metrics (Katsantoni et al., 2023) that we calculated using a custom genome annotation and featureCounts tool (Liao et al., 2014). All samples had median per-base sequencing quality of more than 30 and only a handful of samples had less than 90% of fragments mapped to protein-coding genes; 15 samples with TIN score below 50 were discarded (Supplementary Figure S2A, S4A).

Three of the four pairs of glioblastoma tumor center-periphery samples from the University Hospital Basel clustered as expected in the PCA plot of gene expression (Supplementary Figure S4B), one pair, in which the periphery sample had the lowest TIN score, did not (Supplementary Figure S4A). This clustering was marked by a coherent separation between periphery and tumor center samples by the first principal component (periphery samples were placed left, while tumor center samples were placed right).

ENCODE RBP depletion experiments were performed in two cell lines, K562 and HepG2, and were designed such that the same control samples (116 in total) were used in 1–20 experiments, with the median being eight experiments (Supplementary Table S1). Each control sample was paired with a particular bioreplicate thus forming 58 pairs of bioreplicates. We obtained the first two components from PCA and UMAP analysis of gene expression for these samples and removed pairs for which the Euclidean distance was higher than q75 + 1.5*IQR, where q75 and IQR denote 75% quartile and interquartile range, respectively (Supplementary Figure S4A) in either PCA or UMAP, as we considered these to be outliers. Next, we verified that remaining samples clustered by cell line in PCA and UMAP plots, HepG2 displaying larger variability in comparison to K562 (Supplementary Figure S4B). We then repeated the same procedure with the retained samples, but using PAS scores of proximal poly(A) sites instead of gene expression values as input to PCA and UMAP analysis, again removing bioreplicate outliers (Supplementary Figure S4C) and verifying that the remaining samples clustered by cell line (Supplementary Figure S4D). As a result of these quality controls, 84 control samples, used across 313 depletion experiments, were retained. We further analyzed the usage of 2,953 expressed proximal PAS and removed 1,286 PAS for which PAS scores strongly fluctuated within control samples from HepG2 or K562 cell lines (Supplementary Figure S4E). Finally, in each RBP perturbation experiment, we checked that RBP expression significantly decreased, as expected, upon depletion and the dispersion trend was well captured by DEXseq (Anders et al., 2012); otherwise, the experiment was excluded from the analysis, which limited the data to 215 depletion experiments (119 in K562 and 96 in HepG2 cell lines). The key information from the quality control analysis was appended to the sample list in Supplementary Table S1.

### Statistical analysis

The data were analyzed using python version 3.10.8 and R statistics software version 4.1.3. Quantile regression models were fitted with the statsmodels package. Non-parametric tests were performed with the scipy.stats python package. *p*-values were adjusted for multiple testing using Benjamini–Hochberg approach (Benjamini and Hochberg, 1995), if not specified otherwise. In all figures, the significance levels 0.05, 0.01, and 0.001 are denoted by *, **, and ***, respectively.

### Data and code availability

RNA sequencing data has been deposited to the NCBI BioProject database, under accession PRJNA1060502. Processing scripts are available from the zenodo repository.

## Results

### General patterns of gene expression and APA in GBM subtypes

**FIGURE 1:**
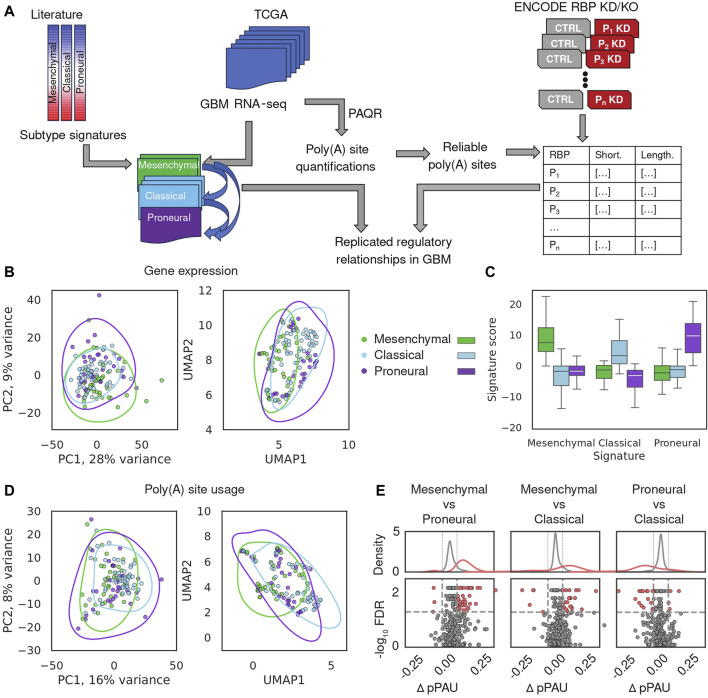
Overview of gene expression and APA in glioblastoma. **(A)** Schematic illustration of the analysis. **(B)** Gene expression-based relationship of TCGA-GBM samples. Sample subtype annotation provided in (Wang et al., 2018) is shown with different colors, in PCA and UMAP representations. Lines represent 75% quartiles of 2D kernel density-estimated joint distributions. Top 987 genes with highest median normalized expression were used as input. **(C)** Distribution of subtype signature scores, calculated with the procedure described in (Phillips et al., 2020), in the 122 TCGA GBM samples stratified by the subtype annotation. **(D)** PCA and UMAP representation of sample relationships based on PAS scores of the proximal poly(A) sites (see Methods). 987 pPAS with defined reliable usage value across all TCGA-GBM samples, were used as input. **(E)** Summary of APA differences between GBM subtypes. Each point is an event, red - significant, gray—not significant in terms of the difference in usage of the proximal PAS between samples of different subtypes (see Methods).

A schematic depiction of our analysis workflow is shown in Figure 1A. We downloaded BAM files for 122 GBM samples from the GDC portal (https://portal.gdc.cancer.gov/) and realigned the reads to retain those that mapped uniquely to the genome and had low duplication rate (see Methods). We then estimated gene expression levels and poly(A) site usage for all potential poly(A) sites in the expressed genes (see Methods). To validate this preliminary analysis, we build on the study of (Wang et al., 2018), who identified genes whose expression defines three main GBM subtypes, namely, proneural, classical and mesenchymal, in order of their presumed malignancy level, and provided the corresponding annotation of TCGA-GBM samples with the most plausible subtype. We estimated expression levels of protein-coding genes across TCGA-GBM samples with our approach (Figure 1B, see Methods) and calculated the subtype signature score for all samples that were already annotated with one of the three subtypes, finding that indeed, the signature score and sample subtype annotation matched those previously reported (Wang et al., 2018) (Figure 1C). The sample separation in the PCA plot was associated with the previously reported degree of transcriptional homogeneity of TCGA-GBM samples, as expected (Supplementary Figure S2B (Wang et al., 2018)). Nevertheless, the separation between subtypes based on gene expression was imperfect, as described in the previous study. This suggested so-far unidentified sources of intratumor heterogeneity, previously confirmed in histopathological, immunohistochemical, and single-cell RNA-seq assays (Wang et al., 2018; Becker et al., 2021). In parallel, we quantified the use of annotated poly(A) sites from the polyAsite database (Herrmann et al., 2020), identifying 987 expressed proximal poly(A) sites that passed stringent criteria of quality (Gruber et al., 2018) across all samples. PCA of poly(A) site usage scores across samples (Figure 1D; Supplementary Figure S2C, see Methods) did not delineate the GBM subtypes, indicating that transcription and polyadenylation are non-redundant mechanisms for regulating gene expression, as observed before (Lianoglou et al., 2013). Finally, we used DEXSeq (Anders et al., 2012) to carry out differential analysis of poly(A) site usage in pairs of subtypes and found that the usage of pPAS is higher in the mesenchymal subtype compared to both classical and proneural (Figure 1E). This indicates a possible association between malignancy and the length of TEs, specifically, relative to the classical subtype, TEs tend to be shortened in the more malignant mesenchymal subtype and lengthened in the less malignant proneural subtype. Thus, our analysis reproduces the proposed classification of GBM subtypes based on mRNA expression and reveals their weak but detectable association with TE shortening and lengthening.

### Association of gene expression and APA with tumor purity and survival

The clinical importance of GBM transcriptional subtypes for guiding therapeutic choices was highlighted by several studies (Zhang et al., 2020). For instance, the addition of Bevacizumab to the first-line treatment provided a progression-free survival benefit only for mesenchymal and proneural tumors, as revealed in a retrospective analysis of biospecimens from patients involved in a clinical study (Sandmann et al., 2015). However, high intratumor heterogeneity of GBM cancers hinders the ability of transcriptional subtypes to predict survival. In addition, transcriptional subtypes were reported to arise from infiltration with immune cells, especially M2 macrophages and neutrophils (Wang et al., 2018), which affect the “tumor purity”. Isoform-based subtyping of GBM was shown before to improve survival prediction compared to gene expression-based subtyping (Pal et al., 2014). We therefore decided to elaborate on this and systematically analyze the association of gene expression and APA with tumor purity and survival.

**FIGURE 2:**
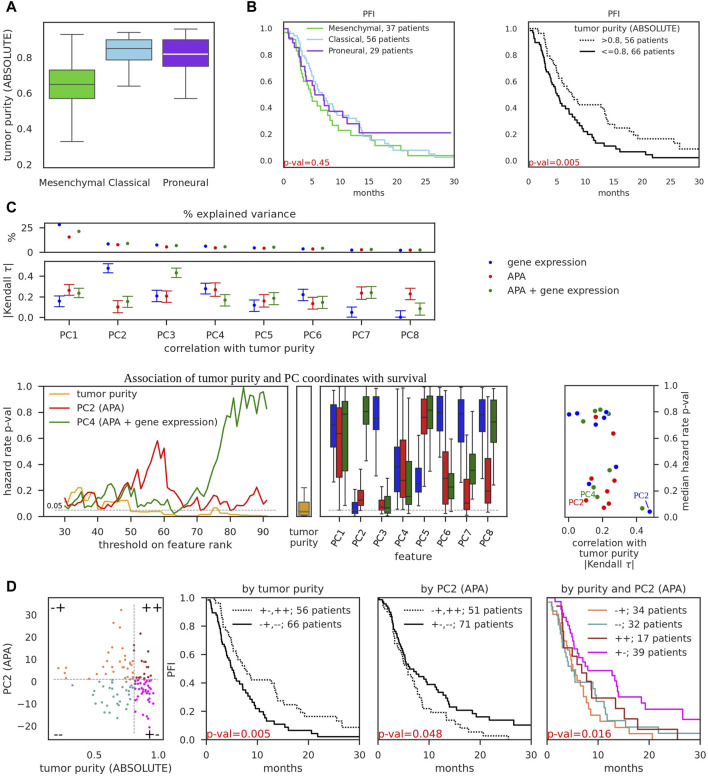
Association of gene expression and APA events with tumor purity and progression-free survival. **(A)** Tumor purity of TCGA-GBM samples of different subtypes, as determined by ABSOLUTE (Carter et al., 2012) (obtained from (Wang et al., 2018)). **(B)** Kaplan-Meier curves of progression-free interval events (PFI) as a function of tumor subtype (left) and tumor purity (right). *p*-values were obtained from the K-sample log-rank hypothesis test of identical survival functions implemented in the python package scikit-survival (Fleming and Harrington, 1981). Survival data was obtained from TCGA-Clinical Data Resource paper (Liu et al., 2018). **(C)** PCA performed on 987 input features of three distinct types: gene expression values (the same as on Figure 1B), pPAS scores (APA, the same as on Figure 1D), and combined, i.e., top 493 pPAS with lowest number of zero scores and 494 genes with highest median expression (totalling to 987 input features, APA + gene expression, see Supplementary Figure S3A). Top: Percentage of variance explained by individual principal components (above) and absolute value of Kendall tau correlation coefficient, calculated between principal component coordinates and tumor purity. 95% bootstrap CI is shown. See Supplementary Figure S3 for additional details and examples. Bottom: left - PC coordinate values and tumor purity values across 122 TCGA-GBM samples were transformed to ranks (in ascending order, i.e., the higher the value - the higher the rank). Thresholds on rank values were iteratively used to separate the dataset into two groups - below and above the threshold, and 2-sample log-rank hypothesis test of identical survival functions was used to obtain the *p*-value for each threshold. These *p*-values (*y*-axis) are shown as a function of threshold (*x*-axis) on tumor purity, PC2 (APA), and PC4 (APA + gene expression) values. Iterations included ranks from 30 to 92 to ensure that threshold-separated groups contain at least 30 samples; middle - boxplot of calculated *p*-values for individual cutoffs on tumor purity; right - boxplots of calculated *p*-values for individual cutoffs on PC coordinates; rightmost panel - scatter of Kendall tau correlation of PFI with tumor purity (*x*-axis, the values were obtained from Panel **(C)**, top subpanel) and median *p*-values for the hazard ratio (*y*-axis, the values were obtained from **(C)**, bottom left subpanels). PC2 (APA), PC4 (APA + gene expression), and PC2 (gene expression) are highlighted. **(D)** Left-most subpanel: 122 TCGA-GBM samples were separated into four groups by putting thresholds on tumor purity value (*x*-axis) and PC2 (APA) value (*y*-axis). Thresholds were selected to minimize *p*-value (maximize significance) for the association with survival (**(C)**, bottom left subpanels). Middle subpanels demonstrate the association of tumor purity-separated groups and PC2-separated groups with progression-free survival. Right-most subpanel demonstrates the association of four-group-separation from the left-most subpanel with progression-free survival.

First, we reproduced the reported decreased tumor purity of the mesenchymal subtype relative to classical and proneural subtypes ((Wang et al., 2018), Figure 2A). Further, we found that while subtypes are weakly associated with progression-free survival (as also reported in (Wang et al., 2018)), tumor purity has strong significant association (Figure 2B). Taken together these results suggest that association of transcriptional subtypes with survival may be solely attributed to tumor purity. We therefore further asked whether we can identify transcriptomic features that are significantly associated with survival but not with tumor purity, as these could provide additional predictive power. We reasoned that while individual transcriptomic events (particular genes or PAS) may not be sufficiently reliable predictors across all tumors, collections of such features constructed would be more statistically robust. Motivated by several previous studies (e.g., (Bair et al., 2006; Shen and Huang, 2006)), we decided to analyze the association of principal components (PC) with survival and tumor purity. Thus, we obtained PC values of TCGA-GBM samples for eight top principal components (Figure 2C, top) from the gene expression and APA PCA conducted above (Figures 1B,D). In addition, we combined gene expression and APA events and also performed PCA (Supplementary Figure S3A). To ensure a fair comparison, we used the same number of input features in the gene-expression, APA, and APA + gene expression-based PCA. We then calculated Kendall Tau coefficients of correlation between each of the PC coordinates with tumor purity (Figure 2C, top; Supplementary Figure S3B). The gene expression-based PC2 component, along which the transcriptional subtypes were separated (Figure 1B) and which explained 9% of variance, exhibited the strongest association with tumor purity, in concordance with observations on Figure 2A. In contrast, the APA-based PC2 component, while explaining a similar ∼8% of APA variance in the dataset, had relatively weak association with tumor purity.

Next, we probed the association of PC components with survival. We decided to employ the same K-sample log-rank hypothesis test of identical survival functions that we used before to analyze the association of transcriptional subtypes with survival (Figure 2B). This technique enables us to directly compare *p*-values of alternative sample groupings. Thus, we transformed PC coordinates and tumor purity values into ranks and iteratively tested the ability of possible rank thresholds to separate the dataset into two groups with survival differences (Figure 2C, bottom left). APA-based PC2 and APA-and-gene expression-based PC4 were among the best-performing features with low *p*-values across many possible thresholds, although still not as strongly predictive for survival as tumor purity. Finally, we juxtaposed Kendall correlation values of PCs with respective median (across different thresholds) *p*-values of PCs which further highlighted APA-based PC2 and APA-and-gene expression-based PC4 as attractive features to distinguish the samples, being strongly associated with survival and weakly associated with tumor purity (Figure 2C, bottom right; Supplementary Figure S3C), thus providing orthogonal information with respect to tumor purity. In comparison, gene expression-based PC2 had even stronger association with survival but also very strong correlation with tumor purity (Supplementary Figure S3B). We further showed that separating the dataset into four groups using thresholds of APA-based PC2 coordinate values and tumor purity allows to obtain statistically significant association with survival (Figure 2D), in large contrast to transcriptional subtype-based separation (Figure 2B, left). As an added value over the already significant association based solely on tumor purity (Figure 2D, middle), two groups stood out as exhibiting clear “intermediate” state between the most malignant (Figure 2D, right, orange curve) and least malignant (Figure 2D, right, magenta curve) groups.

### Identification of RNA-binding protein regulators of polyadenylation

**FIGURE 3:**
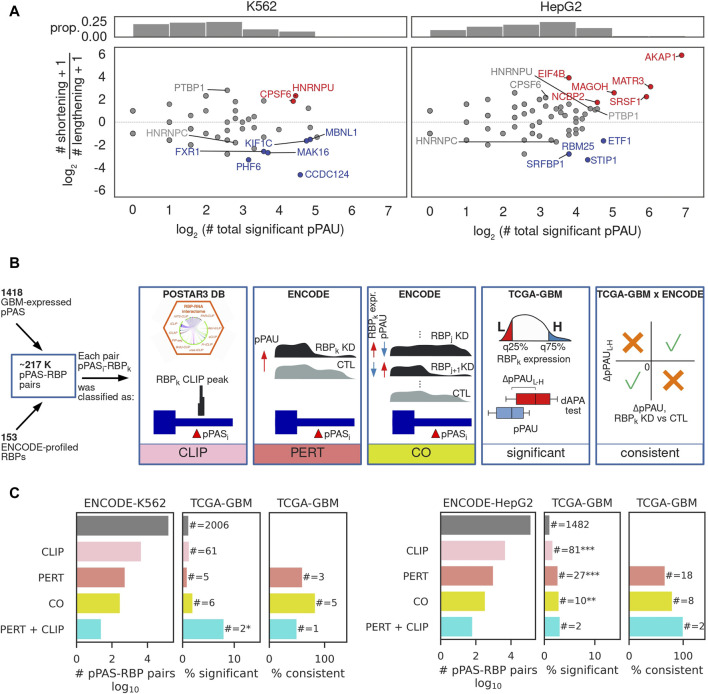
Identification of RBP-regulators of APA. **(A)** Summary of RBP impact on PAS usage in K562 (left) and HepG2 (right) cells. Top panels: histograms of the number of significant pPAS usage changes occurring upon depletion of an RBP. Bottom panel: overall effect of individual RBPs on TE length. *x*-axis indicates the number of significantly changing pPAS upon depletion of the RBP and *y*-axis the impact of the change on TE length (log2 of the ratio of shortening vs lengthening TEs). Each dot corresponds to an RBP whose expression was perturbed. Red/blue highlight RBPs whose depletion leads to predominant shortening/lengthening of TEs, determined from a binomial test of # shortening TEs vs. # shortening TEs+# lengthening TEs, at FDR<10%, gray - no strong preference for the direction of the TE length change. **(B)** Schematic illustration of the type of relationship between a poly(A) site and an RBP. CLIP: pPAS located in terminal exons on which the RBP has been found by crosslinking and immunoprecipitation (CLIP). PERT: pPAS responding to the RBP depletion. CO: pPAS whose usage correlates with the RBP level across all ENCODE perturbations, as determined from a Chi-square test between the numbers of upregulated, downregulated, and non-significant differential RBP expression and dAPA events, at FDR<0.05. Significant: pPAS whose usage is significantly associated with RBP expression in TCGA-GBM data. Consistent: pPAS that respond consistently to RBP perturbation in ENCODE and to changes in RBP level in TCGA-GBM data. In case no regulatory relationship from the ENCODE (PERT, CO) or CLIP data was identified, a pPAS-RBP pair was assigned a category “OTHER”. See the Methods for details. **(C)** Summary of regulatory relationships inferred from ENCODE RBP perturbation data and TCGA-GBM data. Left panel starts from relationships inferred from perturbations in the K562 cell line, right panel from perturbations in the HepG2 cell line.

While APA is extensively studied in cancers where a general trend towards TE shortening relative to matched control tissue has been described (Xia et al., 2014), the regulators driving this pattern of APA in individual cancers are still largely unknown (Gruber and Zavolan, 2019; Masamha, 2023). To reveal such regulators in GBM we adopted an approach that was introduced in (Mironov et al., 2023), where it was used to identify regulators of alternative splicing. The concept is to leverage data from experiments in which individual RNA-binding proteins (RBPs) were depleted to identify RNA processing events that are associated with each RBP, and then select associations that are consistently present (i.e., statistically significant and identical regulatory effect) in patient data. The RBP perturbation data enables a more reliable identification of direct regulatory interactions, as the complexity of gene expression changes is much lower compared to those occurring in cancers. The ENCODE consortium generated RNA sequencing data from K562 and HepG2 cell lines in which individual RBPs (∼200 in total) were depleted by shRNA-mediated knockdown or CRISPR-based knockout. We downloaded the corresponding sequencing files from (Van Nostrand et al., 2020) and after extensive quality control analysis (see Methods) we determined the number of pPAS undergoing a significant change in usage upon each RBP perturbation and also assessed whether the effect of this change is an increase or decrease in the length of the corresponding TEs (Figure 3A). The results revealed 37 RBPs whose depletion is associated with TE shortening or lengthening in at least one of the two cell lines. These included CPSF6, PTBP1 and HNRNPC and HNRNPU, though the magnitude and significance of their effect varied between lines (Supplementary Figure S6). While for 24 (∼65%) of them there is no co-directionality of pPAS usage alterations as measured by cosine similarity (Supplementary Figure S6A), for 11 RBPs the effects were consistent (positive cosine similarity) and only 2 RBPs showed opposite effects (negative cosine similarity). We further asked whether the predominant direction of the effects (shortening vs. lengthening) is consistent between cell lines and found that 19 RBPs showed a consistent pattern (Supplementary Figure S6B), the most consistent being CPSF6 (Martin et al., 2012), PTBP1 (Gruber et al., 2018; Bak et al., 2024), and HNRNPC (Gruber et al., 2016), RBPs that were previously implicated in APA regulation. Thus, our approach identifies some of the well-known regulators of APA, but also several other RBPs whose perturbation consistently impacts the length of TEs or even particular APA events, directly or indirectly.

We next asked whether the pPAS that respond to RBP perturbations in cell lines also show evidence of RBP-dependent expression in GBM samples. To answer this question we implemented the analysis depicted in Figure 3B and characterized the RBP-pPAS associations by the type of evidence supporting them (Figure 3C). Namely, we started from all possible pPAS-RBP associations that could be quantified in both TCGA-GBM and ENCODE RBP perturbation data (see Methods). First, we identified pairs for which there was evidence of direct binding of the RBP to the TE in which the pPAS was located, based on crosslinking and immunoprecipitation data (CLIP) from the POSTAR3 database (Zhao et al., 2022). Next, we identified pPAS that significantly respond to RBP depletion (Figures 3B,C - PERT). Given that RBPs extensively regulate each other’s expression (Leclair et al., 2020), we further checked for correlations of pPAS usage with RBP levels across all ENCODE perturbation experiments, identifying statistically significant associations (Figures 3B,C - CO). The results are summarized in Figure 3C, showing that experimental evidence of pPAS-RBP associations from CLIP and RBP perturbation data enriches pPAS-RBP pairs that are significantly correlated in TCGA-GBM. For most such pairs, the direction of change in pPAS usage inferred from RBP perturbation experiments in ENCODE is consistent with the direction of change associated with fluctuations in RBP expression in the TCGA-GBM data. The complete list of 2,545 RBP-pPAS pairs (130 RBPs, 169 pPAS) significantly correlated in TCGA-GBM, along with their characterization (CLIP, PERT, CO) is provided in Supplementary Table S2.

### Consistent pPAS-RBP associations in TCGA-GBM

**FIGURE 4:**
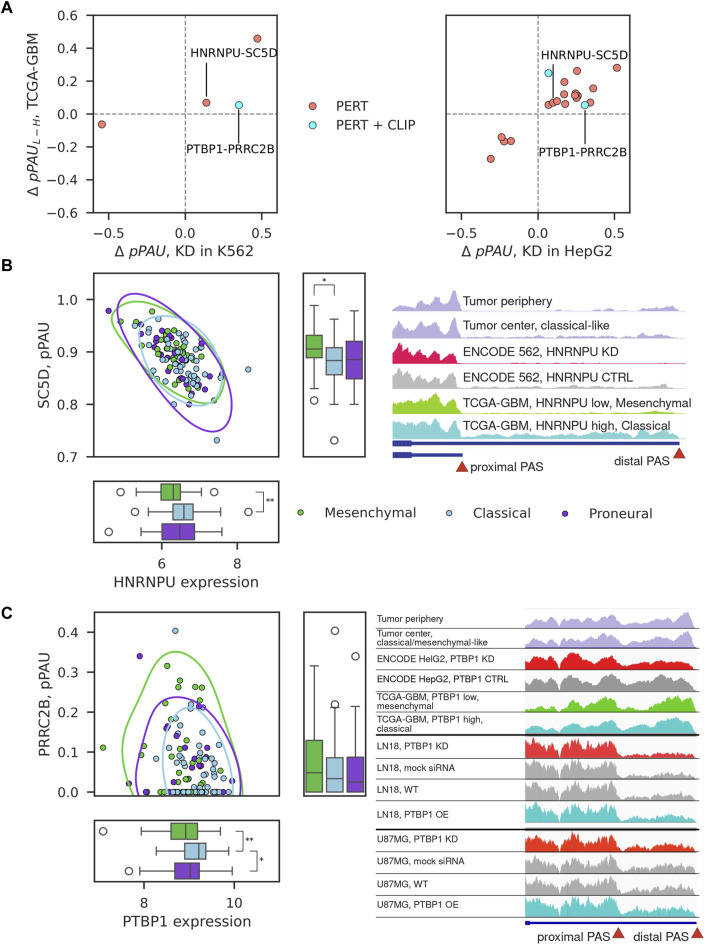
Examples of consistent pPAS-RBP associations. **(A)** Change in pPAS usage in ENCODE vs TCGA-GBM. *x*-axis shows the change upon RBP knockdown (left panel in K562 cells, right panel in HepG2 cells), *y*-axis shows the change when comparing TCGA-GBM samples with low vs high RBP expression. The two associations that were found in both cell lines are highlighted. **(B, C)** Correlation of pPAS usage and RBP expression in TCGA-GBM samples for the two examples that were identified from both cell lines, corresponding to HNRNPU-SC5D and PTBP1-PRRC2B pairs. The subtypes of GBM samples are highlighted with different colors. Significant differences (adjusted *p*-value <0.05) of median RBP expression or PAS usage between TCGA-GBM subtypes are depicted (Mann-Whitney U-test, Bonferroni correction for testing three pairwise comparisons). Non-significant differences are not depicted. Integrative Genome Browser (IGV) screenshot of the coverage of the SC5D terminal exon by RNA-seq reads obtained in different samples (indicated in the different tracks).

We next inspected 22 consistent pPAS-RBP associations characterized as PERT, PERT + CLIP, or CO + PERT (Figure 3C). As shown in Figure 4A, these behaved similarly in RBP perturbation experiments and TCGA-GBM samples with variable expression of the RBP. Two associations were found in both of the analyzed cell lines: HNRNPU-SC5D and PTBP1-PRRC2B. HNRNPU is a splicing factor which recently has been implicated in APA of CD55 in breast cancers (Huang et al., 2023). Its association with sterol-C5-desaturase-like (SC5D) APA appears to be novel. PTBP1 has been implicated in APA in GBM before (Gruber et al., 2018) and here we find that it may regulate APA of PRCC2B, recently found to regulate cell cycle progression in GBM (Jiang et al., 2023). We also found a high correlation of pPAS usage of highlighted events with APA-based PC2 coordinates (Figure 2), with pPAS in SC5D having one of the highest correlation coefficients among all analyzed APA events (Supplementary Figure S7; Supplementary Table S3). Given the link between APA-based PC2 with survival (Figures 2C,D), this suggests that searching for significant RBP-regulated events may also yield novel strong biomarkers of survival in GBM.

While the inter-patient variation is now widely appreciated (e.g., (Friedmann-Morvinski, 2014; Skaga et al., 2019; Becker et al., 2021; Burri and Zavolan, 2021)) and the experimental design usually includes matched non-tumor control tissue, this was not the case for GBM data set in TCGA. For this reason, in the above analysis we compared the patterns of gene expression in cancers considered to have higher/lower malignancy levels. To determine whether a similar regulatory relationship can be detected in tumor-control samples from individual patients we carried out RNA sequencing of four tumor center-periphery sample pairs. With the same analysis of subtype transcriptional signatures (Figure 1C), we were able to assign a dominant type to three of the four sample pairs, one per subtype (Supplementary Figure S4C). We then inspected the coverage of the TE not only in the ENCODE cell lines and TCGA-GBM samples but also in tumor center-periphery sample pairs from our RNA-seq data. The examples of SC5D and PRCC2B are shown in Figures 4B,C. Although, again, the variability between sample pairs is high, we do observe the expected change in pPAS usage between tumor periphery and tumor center, in accordance with the identified tumor subtype. Further, we carried out perturbation experiments (siRNA-mediated KD and overexpression of PTBP1) in two glioblastoma cell lines, U87MG and LN18 (see Methods and Supplementary Figure S8). We focused on PTBP1 because this RBP was already implicated in the regulation of alternative polyadenylation in glioblastomas ((Gruber et al., 2018; Bak et al., 2024)) and, more generally, in carcinogenesis via multitude of pathways (Kim et al., 2021; Huang et al., 2022; Wang et al., 2022; Ye et al., 2024). The experiments validated that the proximal PAS in PRRC2B is used more when PTBP1 levels are low and less when PTBP1 levels are high (Figure 4C). This is in accordance with the predictions from ENCODE HepG2 and K562 experiments and with the observed correlations in TCGA-GBM tumor samples.

Our study provides a blueprint for identifying regulatory relationships between RBPs and individual poly(A) sites in cancers as well as a list of candidates for further investigation. It also highlights the heterogeneity of large, publicly-available data sets and the importance of thorough quality controls to enable distinguishing signal from noise.

## Discussion

Among the many perturbations in gene expression that occur in cancer, the systematic shortening of terminal exons and 3′ untranslated regions (3′UTRs) of mRNAs has been more recently observed (Mayr and Bartel, 2009; Xia et al., 2014). Although 3′UTR shortening was initially associated with increased rate of cell proliferation (Sandberg et al., 2008), another study linked it to the malignant transformation (Mayr and Bartel, 2009), and single cell analyses showed that, in fact, most cells express mRNAs with short 3′UTRs when they are located in a tumor microenvironment (Burri and Zavolan, 2021). How would 3′UTRs become systematically shorter or longer? An initial study associated the increased expression of E2F transcription factors (Elkon et al., 2012), known to regulate the cell cycle (Johnson and Schneider-Broussard, 1998), with increased expression of 3′ end processing factors with the potential consequence of increased processing at coding-region-proximal poly(A) sites. However, 3′UTR shortening is observed upon depletion, not overexpression of the CFIm 3′ end processing factor, indicating that the relationship between the level of 3′ end processing complexes and 3′UTR length is more complex than initially appreciated. Indeed, a very recent study showed that CFIm functions within phase-separated compartments, and that this process is perturbed by the Clk2 kinase-dependent phosphorylation in cancer cells, leading to effective depletion of CFIm in these cells (Liu et al., 2023).

Aside from core 3’ end processing factors, other RBPs have been associated with APA in various conditions, including cancers (Gruber and Zavolan, 2019). Glioblastoma was one of the first cancers where the regulation of polyadenylation has been investigated, and where the CFIm complex was implicated (Masamha et al., 2014). While CFIm perturbations undoubtedly affect the polyadenylation landscape, its contribution to GBM remained unclear, as the inference of altered CFIm expression in GBM relative to normal brain was confounded by the variable degree of RNA degradation among samples (Gruber et al., 2018). In our study, after extensive quality control and subtype classification of the TCGA GBM samples, the differences in expression of CFIm components between the more and less aggressive GBM subtypes were weak in magnitude and statistically non-significant (Supplementary Figure S9). The only exception was CFIm59, also known as CPSF7, but this component of CFIm is not known to affect polyadenylation patterns (Kim et al., 2010; Martin et al., 2012). Another RBP that was linked to APA regulation in GBM is PTBP1, an RBP with increased expression in GBM compared to normal brain 14. Indeed, our analysis revealed one strongly supported association, between PTBP1 and PRRC2B, accompanied by significant GBM subtype-specific PTBP1 expression (Figure 4C, right).

We emphasize that our study is limited by the data currently available. First, we inferred the usage of poly(A) sites from RNA-seq data, with the PAQR package (Gruber et al., 2018), which a recent benchmarking study showed has high accuracy of poly(A) site quantification, while quantifying fewer poly(A) sites compared to other tools (Bryce-Smith et al., 2023). It is likely that direct sequencing of mRNA 3’ ends and subsequent quantification of poly(A) site usage would reveal a larger number of significantly changing sites. Second, we used the ENCODE data set to identify poly(A) sites that respond to perturbations of individual RBPs. While quite extensive, this data set still covers a limited set of RBPs in just two cell lines, and the samples vary in quality, as shown by our quality control analyses. While our analysis shows that significant APA targets tend to respond in the same manner in the two cell lines (Supplementary Figure S6A), the majority of analyzed RBPs do not have consistent effects on APA targets in these cell lines. Thus, it appears that few of the well-characterized RBPs represented in the ENCODE data impact polyadenylation, but it may also be that other, so far unexplored RBPs, impact significantly APA in GBM. Finally, an issue that complicates all analyses based on RNA-seq data is that the inferred regulatory relationships may be indirect. Generally, RBPs with primarily nuclear localization that impact specific TEs are likely to do so at the level of polyadenylation. However, RBPs could induce changes in isoform abundance by regulating other aspects of gene expression, for e.g., mRNA stability. This may make true regulatory relationships yield relatively weak correlations in transcriptomic data from patient samples. Addressing such issues would require additional data sets (e.g., RNA-seq from cell fractions), which are not currently available.

In conclusion, our study provides a workflow for integrating perturbation data from the ENCODE project with data on mRNA isoform expression in cancers (from the TCGA repository) to infer regulatory relationships between RBPs and APA isoforms. We have identified tens of significant relationships that could be further investigated for their contribution to GBM.

## Data Availability

The datasets presented in this study can be found in online repositories. The names of the repository/repositories and accession number(s) can be found below: https://www.ncbi.nlm.nih.gov/, PRJNA1060502 https://doi.org/10.5281/zenodo.13142801, zenodo repository.
