# Supplementary material for: Leveraging multi-omics data to infer regulators of mRNA 3’ end processing in glioblastoma
Source: Front Mol Biosci. 2024 Aug 12;11:1363933. doi: 10.3389/fmolb.2024.1363933 (PMC11345230; doi:10.3389/fmolb.2024.1363933)
Supplement: Supplementary file 4 [file Image1.pdf]

Supplementary figures

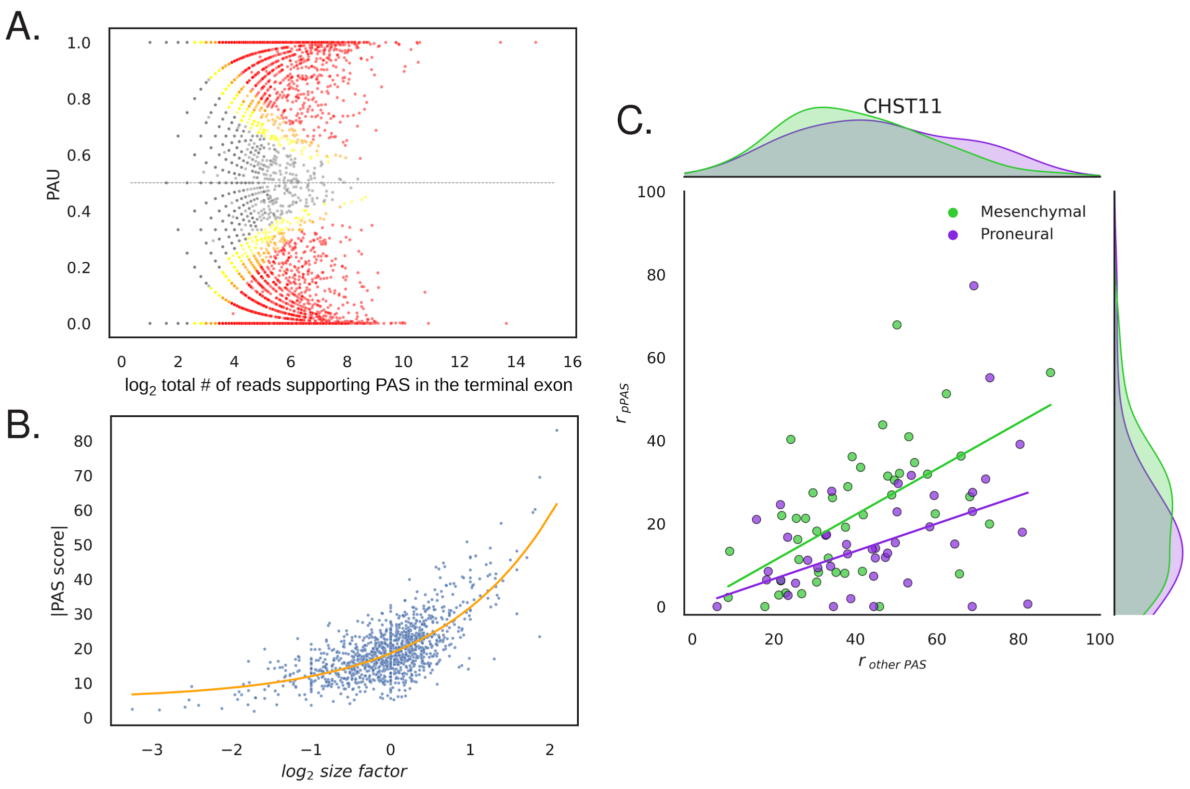

Supplementary figure 1. Quantification of tandem poly(A) sites usage. A. Relationship between the total number of raw reads supporting all PAS in the terminal exon (x-axis, $\log_2$ scale) and PAS usage (y-axis). A random subsample of 10’000 poly(A) sites within all analyzed RNA-seq samples is depicted. Dots are colored by the absolute value of the assigned PAS score (not adjusted for library size, see Methods): less than $-\log_{10}(0.05)$ - grey, from $-\log_{10}(0.05)$ to $-\log_{10}(0.01)$ - yellow, from $-\log_{10}(0.01)$ to $-\log_{10}(0.001)$ - orange, more than $-\log_{10}(0.001)$ - red (see Methods). **B.** An example of PAS score adjustment for library size across all analyzed RNA-seq samples for one randomly selected poly(A) site. The dots represent RNA-seq samples, the absolute values of unadjusted PAS scores are shown on the y-axis, and the $\log_2$ of the estimated size factor (see Methods) on the x-axis. The orange line represents a fitted quantile regression. Residual values above the line are assumed to represent PAS scores not confounded by the library size-driven scaling, while residual values below the line are lower than expected from library-size-driven scaling, and are zeroed to obtain the adjusted PAS score values. **C.** An example of APA analysis for a pPAS in the CHST11 gene and two TCGA-GBM subtypes. The library-size-normalized fragment counts supporting pPAS (y-axis) were modeled as a function of library-size-normalized total fragment counts supporting other PAS in the same TE (x-axis), using quantile regression with a dummy variable corresponding to subtype (see Methods). Predicted values are depicted as lines.

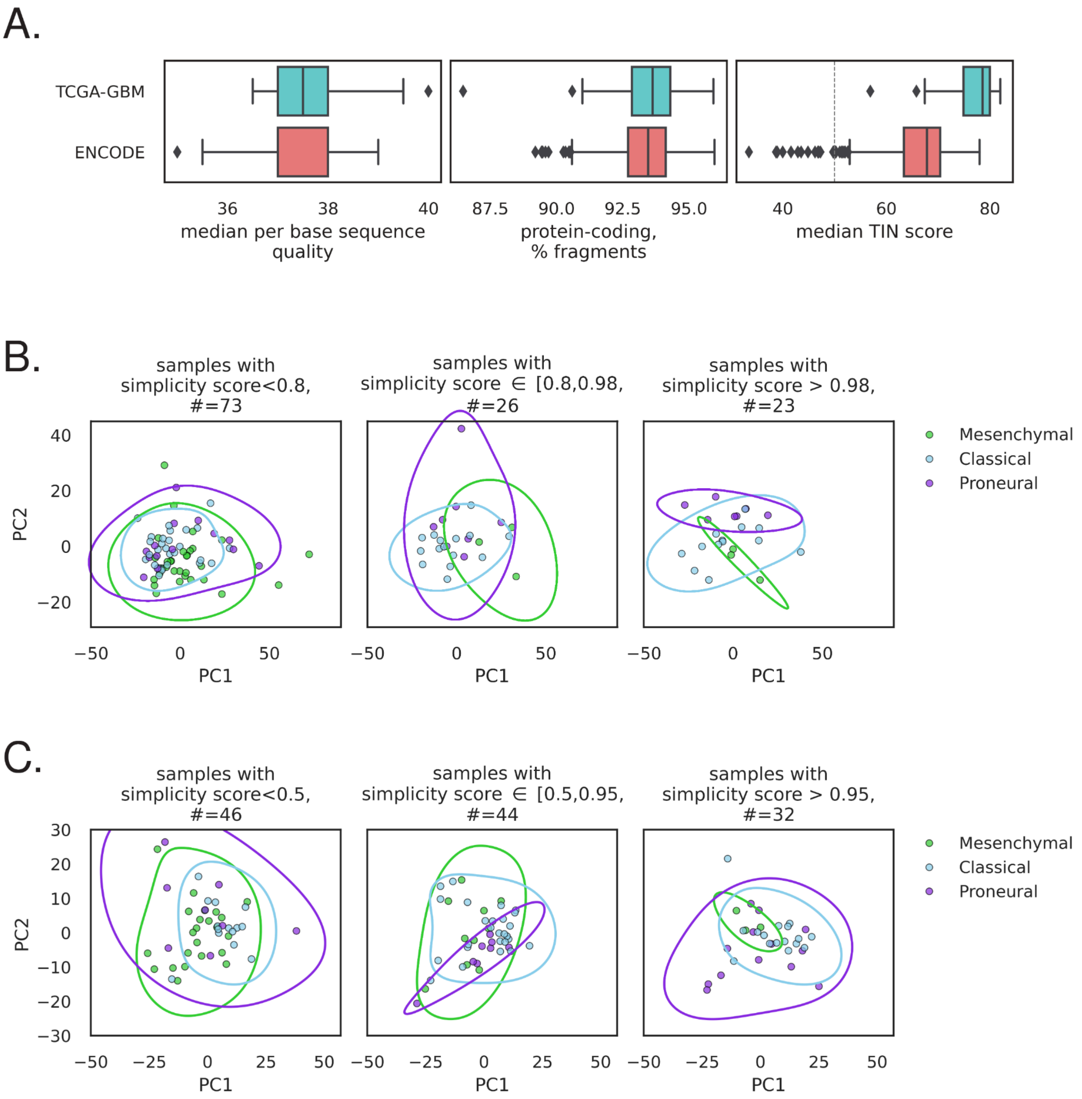

Supplementary figure 2. Quality control, gene expression and APA in TCGA-GBM and ENCODE samples. A. Box plots of quality control metrics calculated for TCGA-GBM and ENCODE RNA-seq samples (see Methods). A TIN score threshold of (50) is shown as a dotted line and was used to select samples of sufficient quality. **B.** Principal component values from the PCA plot depicted at Fig 1B, in three sample cohorts defined by the simplicity score values reported in [1]. Higher simplicity scores correspond to samples with lower intratumor transcriptional heterogeneity. Lines represent 75% quartiles of 2D kernel density-estimated joint distributions. Number of samples in each cohort is shown in titles of the subplots. **C.** The same analysis as in (B), but for PAS scores, based on principal component values from the PCA plot depicted at Fig 1D.

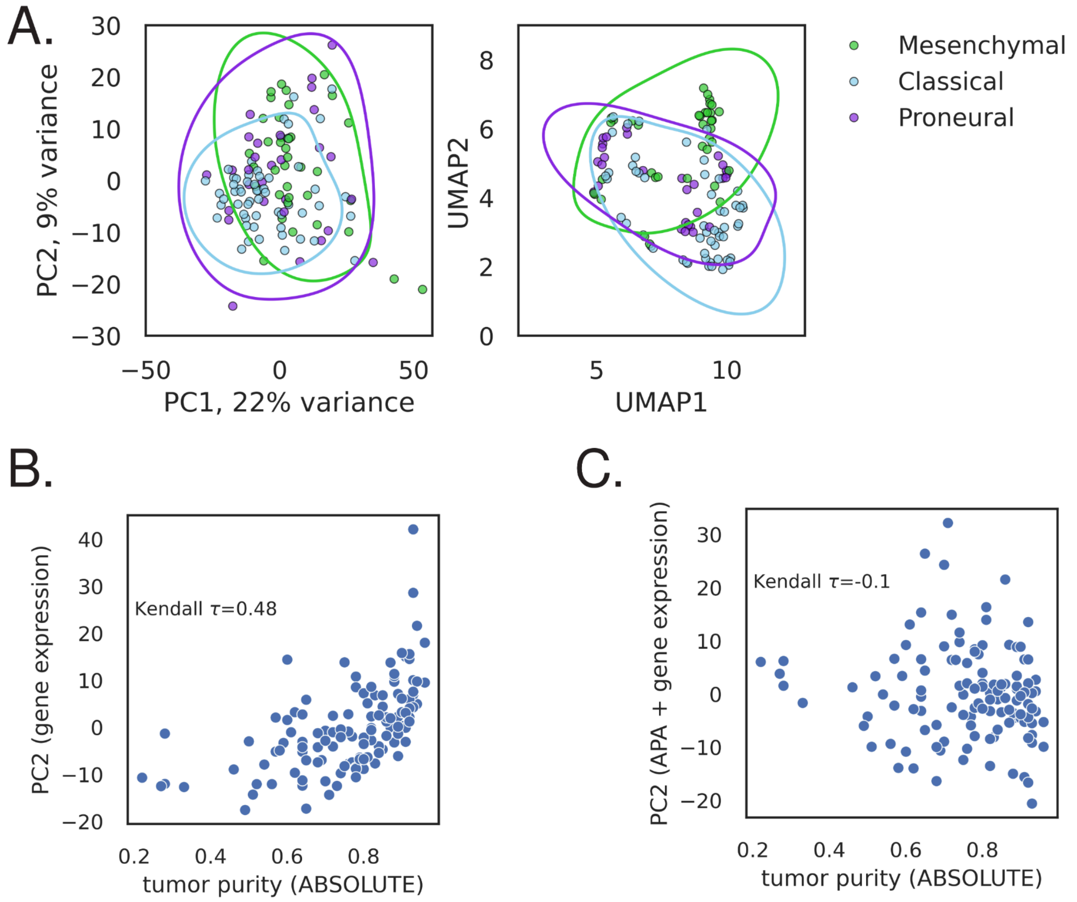

Supplementary figure 3. Association of gene expression and APA events with tumor purity. A. PCA and UMAP on combined APA (top 493 pPAS with lowest number of zero scores across TCGA-GBM samples) and gene expression features (494 genes with highest median expression). In total, 987 input features were used, the same as on Fig. 1B and Fig. 1D. **B.** Kendall correlation of PC2 coordinates from gene expression-based PCA with tumor purity across TCGA-GBM samples. **C.** Kendall correlation of PC2 coordinates from APA-based PCA with tumor purity across TCGA-GBM samples.

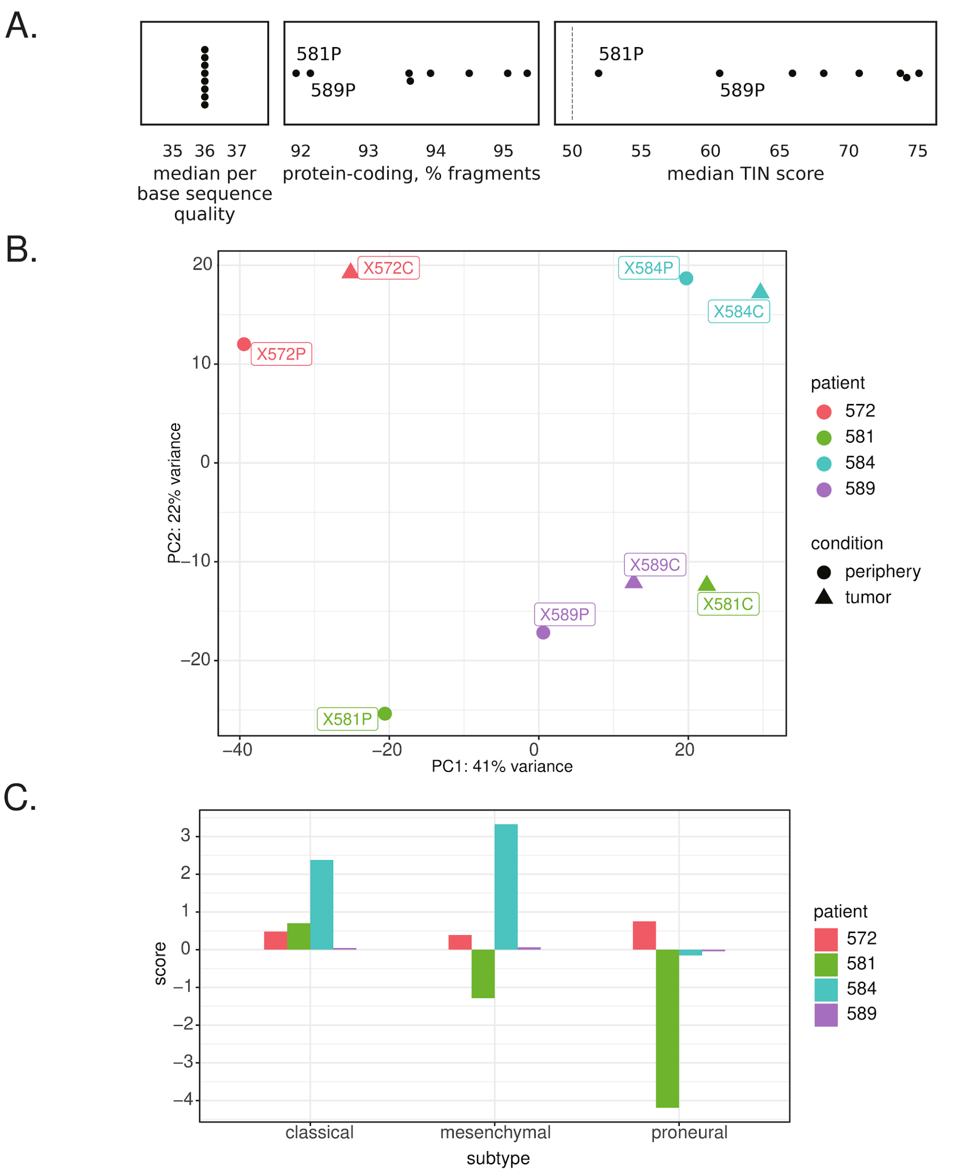

Supplementary figure 4. Gene expression in paired tumor center-periphery samples from GBM patients of the Basel University hospital. **A.** Quality control performed identically to TCGA-GBM and ENCODE samples (Suppl. Fig. 2A). Samples with lowest values of quality measures are highlighted. **B.** PCA plot of gene expression data for the four paired samples. **C.** Barplots showing GBM subtype signature scores for each tumor center sample, calculated in the same way as for TCGA-GBM samples (Fig. 1C).

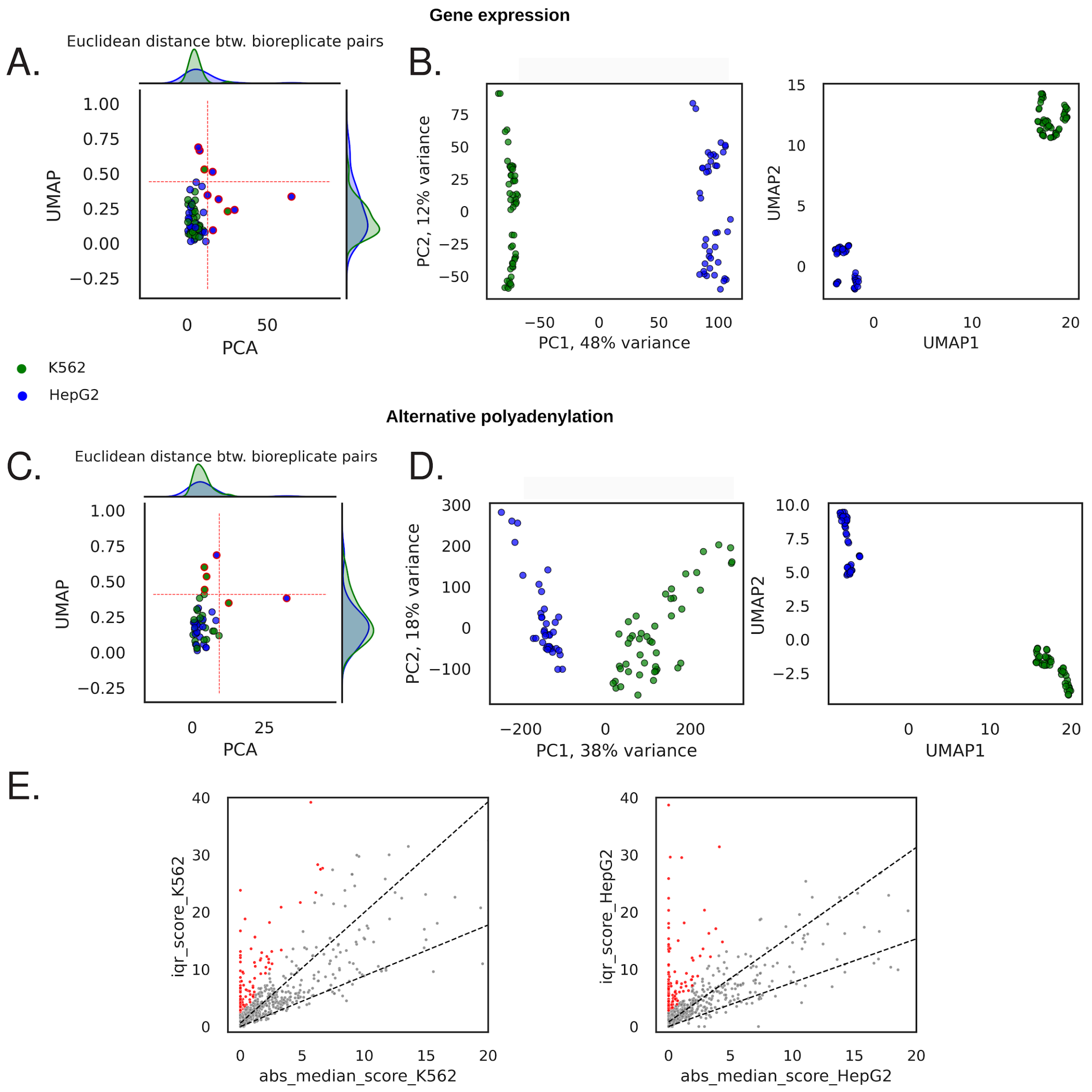

Supplementary figure 5. Quality control of untreated samples from ENCODE RBP depletion experiments. A. Euclidean distances measured between replicates of untreated (control) RNA-seq samples, from the first two principal component values (x-axis) and two UMAP embeddings (y-axis). Green and blue dots denote replicates from K562 and HepG2 cell lines, respectively. Horizontal and vertical red dotted lines represent threshold values, computed as q75+1.5*IQR from distributions of Euclidean distances that were based on PCA (vertical line) or UMAP (horizontal line). q75 and IQR denote 75% quartile and interquartile range. Replicate pairs with Euclidean distances larger than the respective threshold values were considered outliers and were discarded from further analysis. **B.** PCA and UMAP plots of the retained control samples demonstrate consistent separation of samples by cell line. **C, D.** The same analysis as in A and B, but performed based on PAS scores (see Methods) instead of VST-normalized gene expression values. **E.** Identification of pPAS that appear to be outliers in terms of excessively strong fluctuations of their usage scores within untreated samples of K562 (left) and HepG2 (right) cell lines. Dots represent different pPAS. The variability of each pPAS was measured with IQR calculated over samples (y-axis). To cope with the heteroscedasticity of the measurements (higher median

values tend to correspond to higher IQR values), we modeled IQR values as a function of the median PAS absolute score values (x-axis), and fitted two quantile regression equations, one for 75% quartile (q75, top black dotted line) and one for 25% quartile (q25, bottom black dotted line). pPAS whose IQR values were higher than q75+1.5(q75-q25) were considered outliers (colored in red) and were discarded from further analysis.

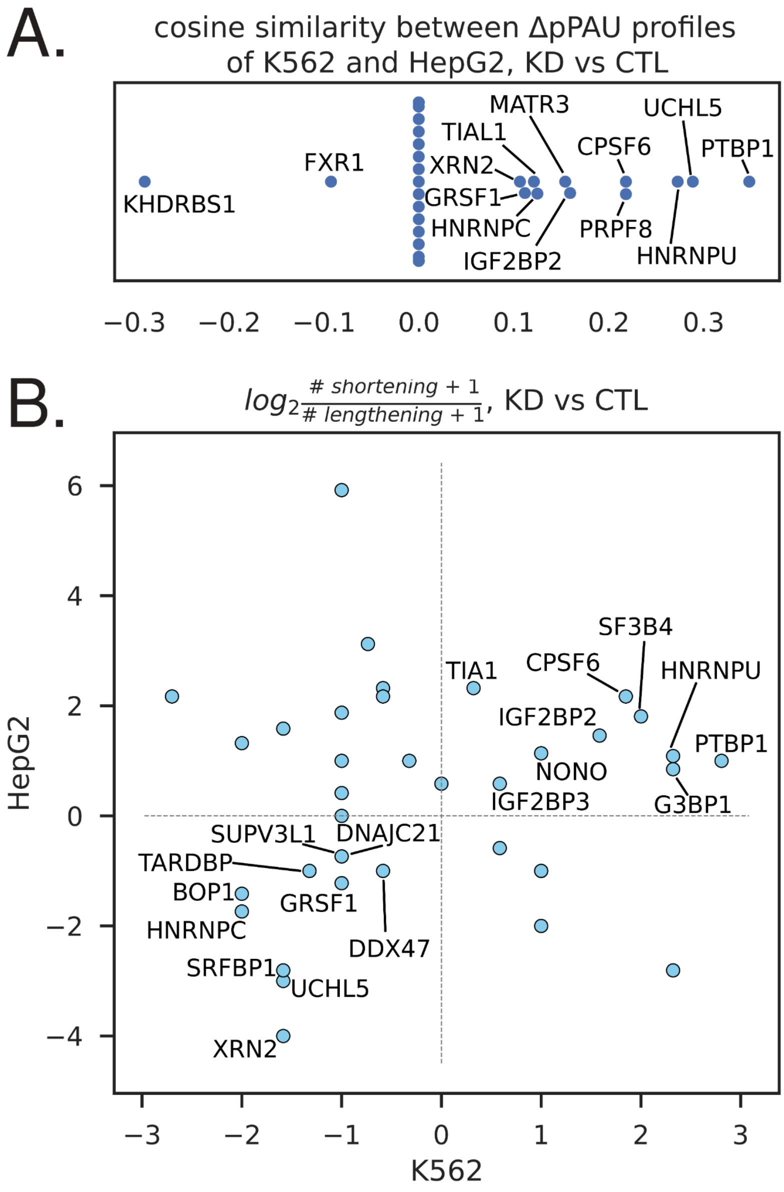

Supplementary figure 6. Similarity of APA changes between HepG2 and K562 ENCODE RBP perturbation experiments. A. 37 RBPs for which at least one significant pPAS with altered usage in KD relative to control in at least one of the cell lines are depicted. For each RBP, the effect for each pPAS usage (up (+1), down (-1), not significant (0), in KD relative to control) in each of the cell lines. Then, for each RBP, cosine similarity on the obtained +1,-1,0 values was calculated between cell lines. RBPs with non-zero cosine similarity values are highlighted. **B.** Overall effects of individual RBP perturbations on TE

length (shortening vs lengthening) is compared between cell lines; corresponds to Fig. 3A. 19 RBPs with consistent direction of the effects on TE length are highlighted.

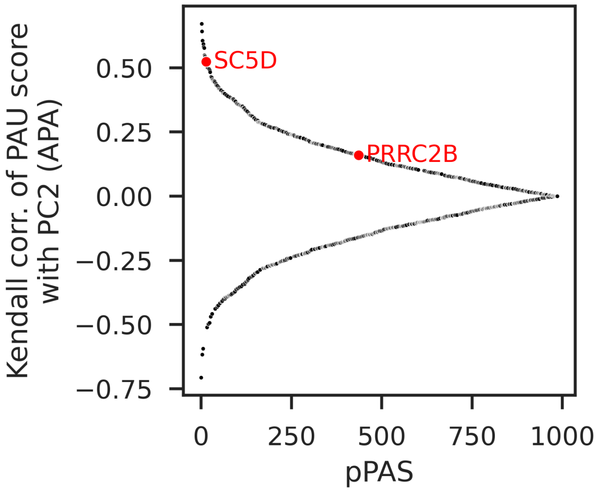

Supplementary figure 7. Kendall correlation coefficients measured between APA-based PC2 coordinate values and each of the pPAS used as input for PCA analysis on Fig. 1D. PAS in SC5D and PRRC2B genes analyzed in Fig. 4, are highlighted. The data source for this figure can be found in Table S3.

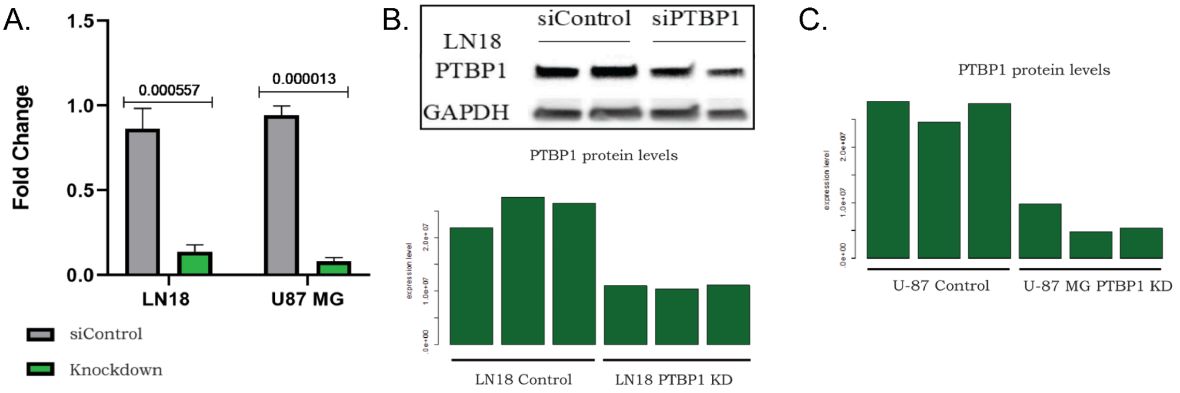

Supplementary figure 8. PTBP1 is knocked down in LN18 and U87 MG cells, by a pool of siRNAs against PTBP1. A. Quantification of PTBP1 mRNA levels in siControl-treated cells (grey bar) and upon PTBP1 knockdown (green bar) in LN18 and U-87 MG using qRT-PCR. **B.** Western blot and quantification of PTBP1 protein levels in Control cells and upon PTBP1 knockdown in LN18. Three biological replicates were generated for each condition. **C.** The same as in B, but for U87MG cell line.

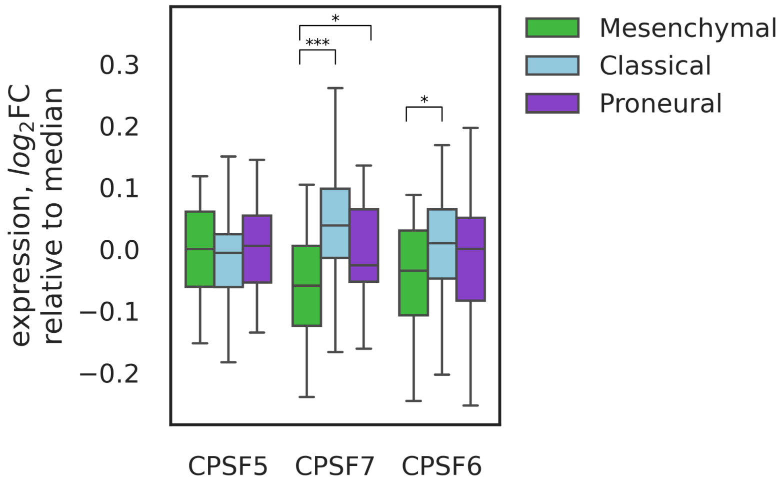

Supplementary figure 9. Variation in expression of CFIm components across TCGA-GBM samples of different subtypes. The expression of CPSF5 aka CFIm25, CPSF6 aka CFIm68 and CPSF7 aka CFIm59 relative to the median over all samples is shown. The results of Mann-Whitney test comparing samples of different subtypes are shown if they yielded a significant p-value. * - p-value < 0.05, *** - p-value < 0.001. P-values were Bonferroni-adjusted for three pairwise comparisons in each gene.

References

1. Wang, Q. *et al.* Tumor Evolution of Glioma-Intrinsic Gene Expression Subtypes Associates with Immunological Changes in the Microenvironment. *Cancer Cell* **33**, 152 (2018).
